# Methyl 2-{4-chloro-2-[5-chloro-2-(2-meth­oxy-2-oxoeth­oxy)benz­yl]phen­oxy}acetate

**DOI:** 10.1107/S160053681202555X

**Published:** 2012-06-13

**Authors:** Michaela Pojarová, Michal Dušek, Zdeňka Sedláková, Emanuel Makrlík

**Affiliations:** a Institute of Physics, AS CR, v.v.i., Na Slovance 2, 182 21 Praha 8, Czech Republic; b Institute of Macromolecular Chemistry, AS CR v.v.i., Heyrovského nám. 2, 16206 Prague 6, Czech Republic; c Faculty of Environmental Sciences, Czech University of Life Sciences, Prague, Kamýcká 129, 165 21 Prague 6, Czech Republic

## Abstract

In the crystal structure of the title compound, C_19_H_18_Cl_2_O_6_, mol­ecules are connected *via* weak C—H⋯π inter­actions into closely packed dimers.

## Related literature

For the synthesis, see: Ertul *et al.* (2009 ▶).
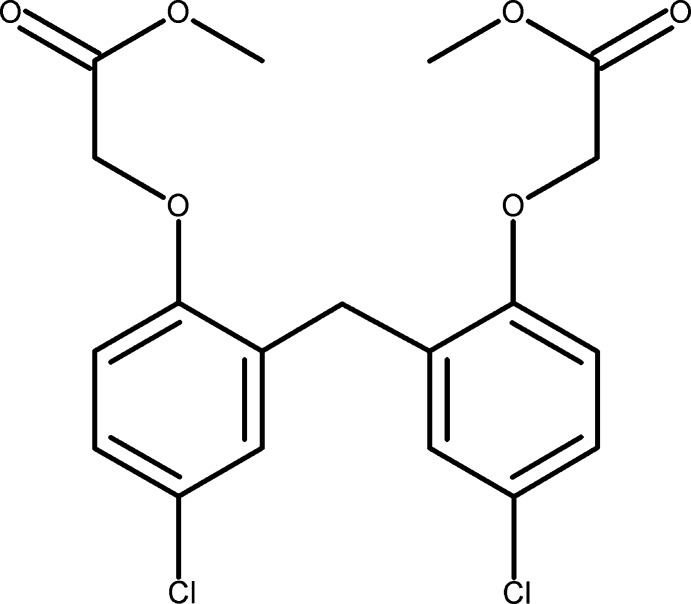


## Experimental

### 

#### Crystal data

C_19_H_18_Cl_2_O_6_
*M* *_r_* = 413.23
Triclinic, 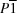

*a* = 7.4727 (6) Å
*b* = 10.4704 (8) Å
*c* = 12.2796 (8) Å
α = 90.384 (6)°
β = 100.716 (6)°
γ = 94.365 (6)°
*V* = 941.08 (12) Å^3^
*Z* = 2
Cu *K*α radiation
μ = 3.41 mm^−1^
*T* = 120 K
0.45 × 0.09 × 0.04 mm

#### Data collection

Agilent Xcalibur Atlas Gemini ultra diffractometer
Absorption correction: multi-scan (*CrysAlis PRO*; Agilent, 2010 ▶) *T*_min_ = 0.258, *T*_max_ = 1.000
8465 measured reflections
3319 independent reflections
2606 reflections with *I* > 2σ(*I*)
*R*_int_ = 0.085

#### Refinement

*R*[*F* ^2^ > 2σ(*F* ^2^)] = 0.080
*wR*(*F* ^2^) = 0.239
*S* = 1.02
3319 reflections
244 parameters
H-atom parameters constrained
Δρ_max_ = 0.92 e Å^−3^
Δρ_min_ = −0.64 e Å^−3^

### 

Data collection: *CrysAlis PRO* (Agilent, 2010 ▶); cell refinement: *CrysAlis PRO*; data reduction: *CrysAlis PRO*; program(s) used to solve structure: *SHELXS97* (Sheldrick, 2008 ▶); program(s) used to refine structure: *SHELXL97* (Sheldrick, 2008 ▶); molecular graphics: *Mercury* (Macrae *et al.*, 2006 ▶) and *ORTEP-3* (Farrugia, 1997 ▶); software used to prepare material for publication: *publCIF* (Westrip, 2010 ▶).

## Supplementary Material

Crystal structure: contains datablock(s) I, global. DOI: 10.1107/S160053681202555X/vm2175sup1.cif

Structure factors: contains datablock(s) I. DOI: 10.1107/S160053681202555X/vm2175Isup2.hkl

Supplementary material file. DOI: 10.1107/S160053681202555X/vm2175Isup3.cml

Additional supplementary materials:  crystallographic information; 3D view; checkCIF report

## Figures and Tables

**Table 1:** Hydrogen-bond geometry (Å, °) *Cg*1 and *Cg*2 are the centroids of the C1–C6 and C8–C13 aromatic rings, respectively.

*D*—H⋯*A*	*D*—H	H⋯*A*	*D*⋯*A*	*D*—H⋯*A*
C12—H12⋯*Cg*1^i^	0.93	2.72	3.500 (3)	142
C17—H17*A*⋯*Cg*2^i^	0.97	2.67	3.450 (3)	138

Symmetry code: (i) 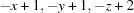.

**Fig. 1.**
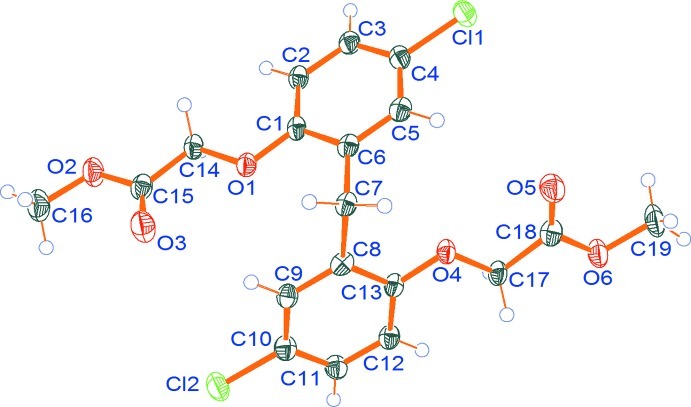
View of the title compound, together with atom-labelling scheme. Displacement ellipsoids are shown at the 50% probability level.

**Fig. 2.**
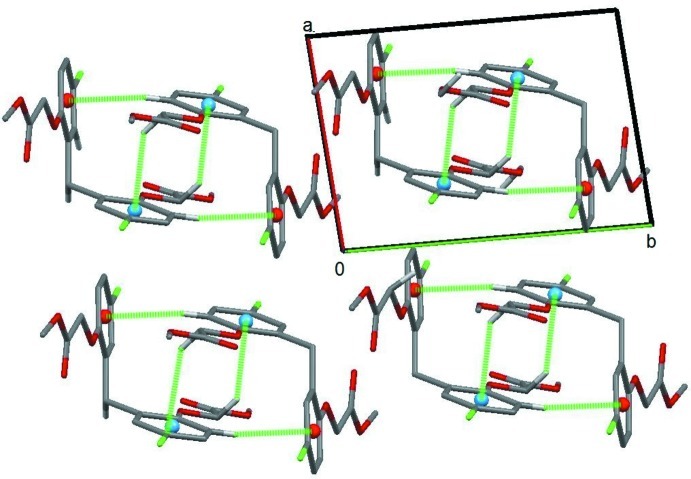
Projection along the c axis with highlighted C—H···π interactions.

## supplementary crystallographic information

### Comment

The title compound is an intermediate in the synthesis of cyclic lactams (Ertul *et al.*, 2009). The molecule consists of two phenyl rings substituted with a chlorine atom in *para* position (Fig. 1). The dihedral angle between the planes of the two aromatic rings is 72.40 (14)°. The arrangement of the molecules is influenced by C—H···π interactions between two neighbouring molecules leading to the formation of closely packed dimers (Table 1, Fig. 2). Due to the presence of aromatic rings, the molecules are also connected *via* system of π–π interactions (Cg1···Cg2^i^: 4.7735 (17) Å; Cg1 and Cg2 are the centroids of rings C1-C6 and C8-C13, respectively; symmetry code: (i) 1 -*x*, 1 - *y*, 2 - *z*).

### Experimental

All chemicals used were purchased from Fluka and used without further purification. The title compound was synthesized by means of the method published by Ertul *et al.* (2009). Crystals were prepared by sublimation (mp. 125 °C, elemental analysis for C_19_H_18_Cl_2_O: calculated C 55.22, H 4.39; found C 55.20, H 4.41).

### Refinement

The H atoms were all located in a difference map and repositioned geometrically. The distance between C and H atoms depends on the carbon atom type and are in a range of 0.93–0.97 Å. The isotropic temperature parameters of hydrogen atoms were calculated as 1.2*U*_eq_ of the parent atom.

### Crystal data

**Table:**

C_19_H_18_Cl_2_O_6_	*Z* = 2
*M**_r_* = 413.23	*F*(000) = 428
Triclinic, *P*1	*D*_x_ = 1.458 Mg m^−^^3^
Hall symbol: -P 1	Cu *K*α radiation, λ = 1.5418 Å
*a* = 7.4727 (6) Å	Cell parameters from 5088 reflections
*b* = 10.4704 (8) Å	θ = 3.7–66.8°
*c* = 12.2796 (8) Å	µ = 3.41 mm^−^^1^
α = 90.384 (6)°	*T* = 120 K
β = 100.716 (6)°	Needle, colourless
γ = 94.365 (6)°	0.45 × 0.09 × 0.04 mm
*V* = 941.08 (12) Å^3^	

### Data collection

**Table:**

Agilent Xcalibur Atlas Gemini ultra diffractometer	3319 independent reflections
Radiation source: Enhance Ultra (Cu) X-ray Source	2606 reflections with *I* > 2σ(*I*)
Mirror monochromator	*R*_int_ = 0.085
Detector resolution: 10.3784 pixels mm^-1^	θ_max_ = 66.9°, θ_min_ = 3.7°
Rotation method data acquisition using ω scans	*h* = −8→8
Absorption correction: multi-scan (*CrysAlis PRO*; Agilent, 2010)	*k* = −11→12
*T*_min_ = 0.258, *T*_max_ = 1.000	*l* = −14→13
8465 measured reflections	

### Refinement

**Table:**

Refinement on *F*^2^	Primary atom site location: structure-invariant direct methods
Least-squares matrix: full	Secondary atom site location: difference Fourier map
*R*[*F*^2^ > 2σ(*F*^2^)] = 0.080	Hydrogen site location: inferred from neighbouring sites
*wR*(*F*^2^) = 0.239	H-atom parameters constrained
*S* = 1.02	*w* = 1/[σ^2^(*F*_o_^2^) + (0.184*P*)^2^] where *P* = (*F*_o_^2^ + 2*F*_c_^2^)/3
3319 reflections	(Δ/σ)_max_ < 0.001
244 parameters	Δρ_max_ = 0.92 e Å^−^^3^
0 restraints	Δρ_min_ = −0.64 e Å^−^^3^

### Special details

**Table:**

Geometry. All e.s.d.'s (except the e.s.d. in the dihedral angle between two l.s. planes) are estimated using the full covariance matrix. The cell e.s.d.'s are taken into account individually in the estimation of e.s.d.'s in distances, angles and torsion angles; correlations between e.s.d.'s in cell parameters are only used when they are defined by crystal symmetry. An approximate (isotropic) treatment of cell e.s.d.'s is used for estimating e.s.d.'s involving l.s. planes.
Refinement. Refinement of *F*^2^ against ALL reflections. The weighted *R*-factor *wR* and goodness of fit *S* are based on *F*^2^, conventional *R*-factors *R* are based on *F*, with *F* set to zero for negative *F*^2^. The threshold expression of *F*^2^ > σ(*F*^2^) is used only for calculating *R*-factors(gt) *etc*. and is not relevant to the choice of reflections for refinement. *R*-factors based on *F*^2^ are statistically about twice as large as those based on *F*, and *R*- factors based on ALL data will be even larger. The H atoms were all located in a difference map, but those attached to carbon atoms were repositioned geometrically. The distance between C and H atoms depends on the carbon atom type and are in a range of 0.93–0.97 Å. The isotropic temperature parameters of hydrogen atoms were calculated as 1.2**U*_eq_ of the parent atom. Unfortunately, the quality of prepared crystals was very low, which lead to the higher *R* factors.

### Fractional atomic coordinates and isotropic or equivalent isotropic displacement parameters (Å^2^)

**Table:**

	*x*	*y*	*z*	*U*_iso_*/*U*_eq_	
C16	0.2502 (6)	1.0126 (5)	1.4550 (4)	0.0432 (11)	
H16A	0.1571	1.0210	1.4984	0.065*	
H16B	0.3059	1.0958	1.4435	0.065*	
H16C	0.3413	0.9607	1.4935	0.065*	
Cl1	0.07945 (13)	0.74416 (9)	0.62990 (8)	0.0353 (4)	
Cl2	0.79884 (14)	0.69660 (9)	1.38850 (8)	0.0377 (4)	
O1	0.3117 (3)	0.8580 (2)	1.1050 (2)	0.0248 (6)	
O4	0.6604 (3)	0.6107 (2)	0.9064 (2)	0.0238 (6)	
O5	0.6164 (4)	0.5956 (3)	0.6838 (2)	0.0384 (7)	
O6	0.7106 (4)	0.3966 (3)	0.6938 (2)	0.0343 (7)	
O3	0.4473 (4)	0.9652 (3)	1.3038 (3)	0.0395 (7)	
O2	0.1699 (4)	0.9529 (3)	1.3497 (2)	0.0361 (7)	
C1	0.2473 (5)	0.8278 (3)	0.9956 (3)	0.0218 (7)	
C13	0.6939 (5)	0.6230 (3)	1.0194 (3)	0.0215 (7)	
C5	0.3291 (5)	0.7943 (3)	0.8183 (3)	0.0254 (8)	
H5	0.4164	0.7883	0.7739	0.030*	
C8	0.6565 (4)	0.7415 (3)	1.0613 (3)	0.0227 (8)	
C4	0.1439 (5)	0.7769 (3)	0.7713 (3)	0.0258 (8)	
C15	0.2866 (5)	0.9364 (3)	1.2822 (3)	0.0281 (8)	
C6	0.3832 (5)	0.8203 (3)	0.9305 (3)	0.0224 (8)	
C17	0.7040 (5)	0.4958 (3)	0.8609 (3)	0.0247 (8)	
H17A	0.6289	0.4241	0.8823	0.030*	
H17B	0.8311	0.4818	0.8885	0.030*	
C7	0.5825 (5)	0.8440 (3)	0.9824 (3)	0.0223 (8)	
H7A	0.6003	0.9256	1.0223	0.027*	
H7B	0.6533	0.8509	0.9237	0.027*	
C3	0.0112 (5)	0.7825 (3)	0.8353 (3)	0.0255 (8)	
H3	−0.1118	0.7694	0.8031	0.031*	
C2	0.0634 (5)	0.8080 (3)	0.9482 (3)	0.0234 (8)	
H2	−0.0249	0.8118	0.9922	0.028*	
C18	0.6699 (5)	0.5054 (3)	0.7362 (3)	0.0275 (8)	
C10	0.7594 (5)	0.6685 (4)	1.2466 (3)	0.0279 (8)	
C14	0.1824 (5)	0.8784 (3)	1.1734 (3)	0.0256 (8)	
H14A	0.1171	0.7979	1.1860	0.031*	
H14B	0.0945	0.9360	1.1381	0.031*	
C11	0.7946 (5)	0.5516 (3)	1.2058 (3)	0.0275 (8)	
H11	0.8394	0.4887	1.2543	0.033*	
C12	0.7631 (5)	0.5283 (3)	1.0930 (3)	0.0251 (8)	
H12	0.7876	0.4499	1.0654	0.030*	
C9	0.6897 (5)	0.7628 (3)	1.1747 (3)	0.0239 (8)	
H9	0.6654	0.8408	1.2033	0.029*	
C19	0.6900 (7)	0.3897 (5)	0.5742 (4)	0.0423 (10)	
H19A	0.7222	0.3076	0.5522	0.063*	
H19B	0.7688	0.4560	0.5501	0.063*	
H19C	0.5655	0.4008	0.5410	0.063*	

### Atomic displacement parameters (Å^2^)

**Table:**

	*U*^11^	*U*^22^	*U*^33^	*U*^12^	*U*^13^	*U*^23^
C16	0.045 (2)	0.054 (3)	0.033 (2)	0.005 (2)	0.0143 (19)	−0.012 (2)
Cl1	0.0456 (6)	0.0380 (6)	0.0222 (6)	0.0074 (4)	0.0048 (4)	−0.0001 (4)
Cl2	0.0541 (7)	0.0351 (6)	0.0231 (6)	0.0083 (4)	0.0034 (4)	−0.0029 (4)
O1	0.0295 (13)	0.0248 (13)	0.0223 (14)	0.0046 (10)	0.0094 (10)	−0.0002 (10)
O4	0.0349 (13)	0.0184 (12)	0.0202 (13)	0.0044 (10)	0.0096 (10)	0.0001 (10)
O5	0.0581 (18)	0.0313 (15)	0.0285 (16)	0.0131 (13)	0.0108 (13)	0.0047 (12)
O6	0.0534 (17)	0.0296 (14)	0.0231 (15)	0.0105 (12)	0.0127 (12)	−0.0013 (11)
O3	0.0335 (16)	0.0513 (18)	0.0335 (17)	−0.0050 (13)	0.0101 (12)	−0.0115 (14)
O2	0.0357 (14)	0.0468 (16)	0.0280 (16)	0.0029 (12)	0.0123 (11)	−0.0084 (12)
C1	0.0345 (18)	0.0108 (15)	0.0220 (18)	0.0019 (13)	0.0098 (14)	0.0007 (13)
C13	0.0274 (16)	0.0176 (16)	0.0218 (18)	0.0018 (13)	0.0104 (13)	0.0025 (13)
C5	0.0326 (19)	0.0175 (16)	0.028 (2)	0.0039 (14)	0.0109 (15)	0.0040 (14)
C8	0.0233 (16)	0.0169 (16)	0.030 (2)	0.0003 (13)	0.0097 (14)	0.0040 (14)
C4	0.040 (2)	0.0153 (16)	0.0232 (19)	0.0047 (14)	0.0062 (15)	0.0002 (14)
C15	0.038 (2)	0.0222 (18)	0.027 (2)	0.0056 (15)	0.0122 (16)	0.0025 (15)
C6	0.0305 (17)	0.0118 (15)	0.0266 (19)	0.0029 (13)	0.0092 (14)	0.0021 (13)
C17	0.0327 (18)	0.0186 (16)	0.024 (2)	0.0036 (14)	0.0083 (15)	−0.0041 (14)
C7	0.0289 (18)	0.0148 (15)	0.0255 (19)	0.0025 (13)	0.0104 (14)	0.0029 (14)
C3	0.0328 (18)	0.0183 (16)	0.026 (2)	0.0039 (14)	0.0069 (15)	0.0003 (14)
C2	0.0288 (17)	0.0184 (16)	0.0246 (19)	0.0006 (13)	0.0095 (14)	−0.0005 (14)
C18	0.0324 (19)	0.0209 (18)	0.031 (2)	−0.0004 (14)	0.0111 (15)	−0.0014 (15)
C10	0.0318 (18)	0.0253 (18)	0.027 (2)	0.0013 (15)	0.0075 (15)	−0.0006 (15)
C14	0.0319 (18)	0.0243 (18)	0.0230 (19)	0.0023 (14)	0.0116 (14)	−0.0010 (14)
C11	0.0350 (19)	0.0246 (18)	0.024 (2)	0.0042 (15)	0.0068 (15)	0.0039 (15)
C12	0.0323 (18)	0.0172 (16)	0.027 (2)	0.0026 (14)	0.0091 (15)	0.0004 (14)
C9	0.0261 (17)	0.0204 (16)	0.0256 (19)	0.0001 (13)	0.0070 (14)	−0.0020 (14)
C19	0.065 (3)	0.042 (2)	0.023 (2)	0.010 (2)	0.0153 (19)	−0.0062 (18)

### Geometric parameters (Å, º)

**Table:**

C16—O2	1.437 (6)	C8—C7	1.517 (5)
C16—H16A	0.9600	C4—C3	1.380 (5)
C16—H16B	0.9600	C15—C14	1.513 (6)
C16—H16C	0.9600	C6—C7	1.508 (5)
Cl1—C4	1.738 (4)	C17—C18	1.510 (5)
Cl2—C10	1.732 (4)	C17—H17A	0.9700
O1—C1	1.367 (4)	C17—H17B	0.9700
O1—C14	1.419 (4)	C7—H7A	0.9700
O4—C13	1.368 (4)	C7—H7B	0.9700
O4—C17	1.409 (4)	C3—C2	1.386 (5)
O5—C18	1.198 (5)	C3—H3	0.9300
O6—C18	1.328 (5)	C2—H2	0.9300
O6—C19	1.449 (5)	C10—C11	1.382 (5)
O3—C15	1.196 (5)	C10—C9	1.394 (5)
O2—C15	1.330 (5)	C14—H14A	0.9700
C1—C2	1.388 (5)	C14—H14B	0.9700
C1—C6	1.411 (5)	C11—C12	1.377 (5)
C13—C8	1.406 (5)	C11—H11	0.9300
C13—C12	1.409 (5)	C12—H12	0.9300
C5—C6	1.381 (5)	C9—H9	0.9300
C5—C4	1.394 (6)	C19—H19A	0.9600
C5—H5	0.9300	C19—H19B	0.9600
C8—C9	1.382 (5)	C19—H19C	0.9600
			
O2—C16—H16A	109.5	C6—C7—H7A	108.6
O2—C16—H16B	109.5	C8—C7—H7A	108.6
H16A—C16—H16B	109.5	C6—C7—H7B	108.6
O2—C16—H16C	109.5	C8—C7—H7B	108.6
H16A—C16—H16C	109.5	H7A—C7—H7B	107.6
H16B—C16—H16C	109.5	C4—C3—C2	119.2 (3)
C1—O1—C14	117.9 (3)	C4—C3—H3	120.4
C13—O4—C17	117.0 (3)	C2—C3—H3	120.4
C18—O6—C19	116.1 (3)	C3—C2—C1	120.1 (3)
C15—O2—C16	114.8 (3)	C3—C2—H2	119.9
O1—C1—C2	124.3 (3)	C1—C2—H2	119.9
O1—C1—C6	114.9 (3)	O5—C18—O6	125.4 (4)
C2—C1—C6	120.8 (3)	O5—C18—C17	126.2 (3)
O4—C13—C8	115.2 (3)	O6—C18—C17	108.4 (3)
O4—C13—C12	124.9 (3)	C11—C10—C9	120.6 (4)
C8—C13—C12	119.9 (3)	C11—C10—Cl2	119.4 (3)
C6—C5—C4	120.2 (3)	C9—C10—Cl2	119.9 (3)
C6—C5—H5	119.9	O1—C14—C15	107.4 (3)
C4—C5—H5	119.9	O1—C14—H14A	110.2
C9—C8—C13	119.0 (3)	C15—C14—H14A	110.2
C9—C8—C7	120.9 (3)	O1—C14—H14B	110.2
C13—C8—C7	120.1 (3)	C15—C14—H14B	110.2
C3—C4—C5	121.3 (4)	H14A—C14—H14B	108.5
C3—C4—Cl1	119.4 (3)	C12—C11—C10	119.8 (3)
C5—C4—Cl1	119.3 (3)	C12—C11—H11	120.1
O3—C15—O2	125.2 (4)	C10—C11—H11	120.1
O3—C15—C14	125.8 (3)	C11—C12—C13	120.2 (3)
O2—C15—C14	109.0 (3)	C11—C12—H12	119.9
C5—C6—C1	118.4 (3)	C13—C12—H12	119.9
C5—C6—C7	121.1 (3)	C8—C9—C10	120.6 (3)
C1—C6—C7	120.4 (3)	C8—C9—H9	119.7
O4—C17—C18	108.6 (3)	C10—C9—H9	119.7
O4—C17—H17A	110.0	O6—C19—H19A	109.5
C18—C17—H17A	110.0	O6—C19—H19B	109.5
O4—C17—H17B	110.0	H19A—C19—H19B	109.5
C18—C17—H17B	110.0	O6—C19—H19C	109.5
H17A—C17—H17B	108.3	H19A—C19—H19C	109.5
C6—C7—C8	114.7 (3)	H19B—C19—H19C	109.5

### Hydrogen-bond geometry (Å, º)

Cg1 and Cg2 are the centroids of the C1–C6 and C8–C13 aromatic rings, respectively.

**Table:**

*D*—H···*A*	*D*—H	H···*A*	*D*···*A*	*D*—H···*A*
C12—H12···*Cg*1^i^	0.93	2.72	3.500 (3)	142
C17—H17*A*···*Cg*2^i^	0.97	2.67	3.450 (3)	138

Symmetry code: (i) −*x*+1, −*y*+1, −*z*+2.

## References

[bb1] Agilent (2010). *CrysAlis PRO.* Agilent Technologies, Yarnton, England.

[bb2] Ertul, S., Tombak, A. H., Bayrakci, M. & Merter, O. (2009). *Acta Chim. Slov.* **56**, 878–884.

[bb3] Farrugia, L. J. (1997). *J. Appl. Cryst.* **30**, 565.

[bb4] Macrae, C. F., Edgington, P. R., McCabe, P., Pidcock, E., Shields, G. P., Taylor, R., Towler, M. & van de Streek, J. (2006). *J. Appl. Cryst.* **39**, 453–457.

[bb5] Sheldrick, G. M. (2008). *Acta Cryst.* A**64**, 112–122. 10.1107/S0108767307043930 18156677

[bb6] Westrip, S. P. (2010). *J. Appl. Cryst.* **43**, 920–925.

